# Bifocal juvenile papillomatosis as a marker of breast cancer: A case report and review of the literature

**DOI:** 10.3892/ol.2014.2600

**Published:** 2014-10-10

**Authors:** TONG WANG, YA-QING LI, HONG LIU, XI-LIN FU, SHOU-CHING TANG

**Affiliations:** 1 Second Department of Breast Cancer, Tianjin Medical University Cancer Institute and Hospital, Ministry of Education, Tianjin 300060, P.R. China; 2 Department of Breast Cancer Pathology and Research Laboratory, National Clinical Research Center for Cancer, Key Laboratories of Breast Cancer Prevention and Therapy, Tianjin Medical University Cancer Institute and Hospital, Ministry of Education, Tianjin 300060, P.R. China; 3 Division of Hematology and Oncology, Georgia Regents University Cancer Center, Augusta, GA 30912, USA

**Keywords:** juvenile papillomatosis, diagnosis and treatment, breast cancer risk

## Abstract

Juvenile papillomatosis (JP), also termed Swiss cheese disease, is a rare and benign type of proliferative breast tumor that is specifically observed in children and adolescents. The majority of JP patients are Caucasian and exhibit a single breast mass. The current report presents an unusual case of bifocal JP in an 11-year-old Chinese female. The patient presented with a slow-growing palpable mass in the upper outer quadrant of the left breast. Ultrasonography identified a further impalpable lesion in the lower outer quadrant of the ipsilateral breast. The preoperative clinical diagnosis of the two masses was fibroadenoma, however, following complete excision of the two tumors, histopathology revealed JP. Furthermore, the patient had a family history of breast cancer. The current report describes a review of the literature regarding the presentation, pathology, diagnosis, and treatment of JP and its association with breast carcinoma. In the current case, JP was associated with an increased risk of breast cancer in the patient, as well as the patient’s elder female relatives; therefore, a more thorough medical follow-up may prove prudent for those individuals with a high risk of developing breast cancer.

## Introduction

Juvenile papillomatosis (JP), also termed Swiss cheese disease, of the breast is a rare and benign disease, which predominantly occurs in females aged <30 years. JP is often preoperatively diagnosed as fibroadenoma due to similarities in the clinical manifestations. However, ductal papillomatosis and cysts are dominant microscopic features that are distinct to JP (1–3). Since JP was first presented by Rosen *et al* (1) in 1980, ~400 cases have been reported, with the majority observed in Caucasian patients and rare cases occurring in patients of Asian origin (4). A family history of breast cancer (in first- or second-degree relatives) has been identified in 26–58% of JP patients (5–8) and breast carcinomas coexisting with JP lesions have been observed in certain cases (1,5,7). A long-term follow-up study demonstrated that certain patients developed breast cancer eight-nine years subsequent to the diagnosis of JP (8); therefore, JP patients appear to have an increased risk of developing breast cancer. The current report presents a case of bifocal JP in an 11-year-old Chinese female and describes a review of the literature. Furthermore, the diagnosis and treatment of JP, as well as the association between JP and breast carcinoma are presented. Written informed consent was obtained from the patient’s family.

## Case report

In January 2010, an 11-year-old Chinese female was admitted to the Second Department of Breast Cancer, Tianjin Medical University Cancer Institute and Hospital (Tianjin, China)presenting with a tender mass in the left breast, which had initially been identified three months previously and had gradually enlarged. The patient had not experienced menarche. The patient’s grandmother was diagnosed with breast cancer at 52 years of age and the patient’s mother had required a lumpectomy for a breast fibroadenoma at 16 years of age, and both individuals have been cured. No history of hormonal or other teratogenic agent use was recorded in the patient or in the mother during pregnancy. Upon physical examination the patient appeared healthy with bilateral normal development of the breasts. A firm, mobile, poorly-circumscribed, tender mass was identified in the upper outer quadrant of the left breast, 1 cm from the areola (size, 3×3×2 cm). The nipple and areola were normal with no nipple discharge and no swollen axillary lymph nodes were observed.

Ultrasonography of the breasts revealed that the palpable mass was poorly defined, irregularly-shaped and inhomogeneous with blood-flow signals. Furthermore, two additional, smaller, impalpable lumps were identified. The first lesion was located in the lower outer quadrant of the left breast (size, 0.9×0.5×1.0 cm) and did not exhibit subcutaneous association with the primary lesion. The second lesion was identified in the upper outer quadrant of the right breast (size, 0.6×0.4×0.4 cm). The two masses were well-circumscribed, regularly shaped and hypoechoic. Ultrasonography determined the three masses as fibroadenomas. A mammography was not performed due to the young age of the patient and no abnormalities were identified during blood tests.

**Figure 1:**
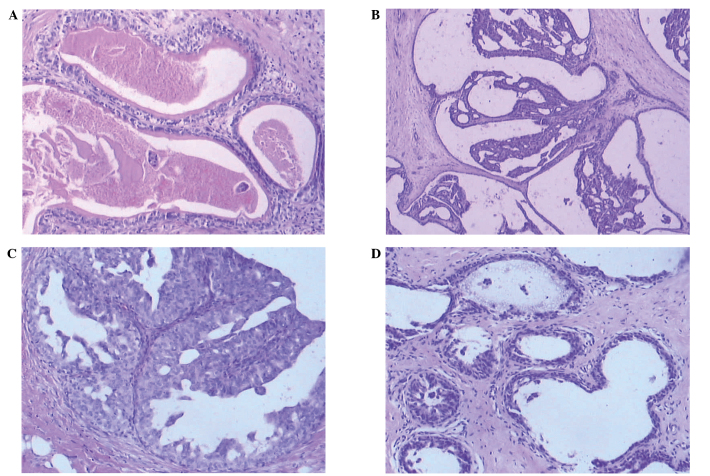
Histopathological findings in the lesions of the left breast by hematoxylin and eosin staining. The larger tumor (size, 2.4×1.5×1.3 cm) exhibited: (A) Multiple dilated ducts containing inspissated secretions and foamy cells (magnification, ×100); (B) intracystic papillary epithelial proliferation (magnification, ×40); and (C) apocrine metaplasia (magnification, ×100). (D) The smaller tumor (size, 1.1×0.7×0.6 cm) exhibited dilated ducts and cysts (magnification, ×40).

The patient and the patient’s parents selected medical follow-up examinations for the mass in the right breast, rather than surgery or a core needle biopsy; however, the two lesions in the left breast were completely excised. One tumor mass measured 2.4×1.5×1.3 cm and the other tumor mass measured 1.1×0.7×0.6 cm. The two lesions were gray, well-demarcated, firm and exhibited no visible cysts, which was consistent with the diagnosis of fibroadenoma, as determined by ultrasound. Furthermore, upon microscopic examination, the two lesions demonstrated similar features. However, histopathology revealed multiple dilated ducts containing inspissated secretions and foamy cells, as well as intracystic papillary epithelial proliferation with apocrine metaplasia (Fig. 1). Thus, the diagnosis of JP of the breast was established. The JP was bifocal as the two lesions were in different quadrants of the breast and were not subcutaneously associated.

Regular patient follow-up, by physical examination and ultrasonography of the breasts, is ongoing, and to date has been conducted for 48 months. No local recurrence or malignant change occurred and ultrasonography of the mass in the right breast exhibited no change. Further follow-up did not indicate the development of new breast disorders among the patient’s relatives.

## Discussion

Papillomatosis has an average of age of onset of 40 years and is associated with a relative risk for breast cancer development in adults (3). In 1980, Rosen *et al* (1) reported 37 cases of papillomatosis in young females with a mean age of 19 years (range, 10–44 years), and defined this novel disease as JP due to its clinical and microscopic features. Thus far, ~400 cases of JP have been reported, the majority in Caucasian females aged <30 years at the time of diagnosis. The present study identified 10 cases of JP in males to date by conducting a search of the English literature using PubMed (http://www.ncbi.nlm.nih.gov/pubmed) (9–11). Cases of JP in Asian individuals were rare and, thus, fewer reports exist. Whether the difference in incidence between Caucasian and Asian populations is due to genetic or environmental factors remains unclear.

The typical manifestation of JP is a unifocal tumor, commonly located in the upper outer quadrant or outer half of the breast, and is firm, well-circumscribed, mobile, painless and generally measures <3 cm in diameter (5). Reports of bloody nipple discharge were unusual (3,5,12). When the clinical diagnosis was determined prior to surgery, it was typically fibroadenoma. Mammography is not routinely recommended for diagnosis or follow-up in females <35 years; however, the few reported mammographic findings regarding JP revealed a well-circumscribed homogeneous opacity, which is similar to that observed in fibroadenomas and cysts (13). Ultrasonography is the preferred imaging technique for JP patients as it facilitates with the differentiation between JP and similar cystic lesions, fibroadenomas, phyllodes tumors, intracystic papillomas and breast cancer (14). Sonographically, the JP lesion presented as a poorly-defined heterogeneous mass with various small, round, echo-free areas, predominantly observed close to the border of the lesion (15). Microscopically, the typical histopathological features are duct papillomatosis with or without epithelial atypia, apocrine and non-apocrine cysts, duct stasis and sclerosing adenosis (1). Papillomatosis and cysts are the dominant diagnostic criteria of JP. A case report describing the fine-needle aspiration cytology of JP (16) revealed the tumor to be comprised of sheets of hyperplastic breast epithelium with areas resembling fibroadenoma, and containing macrophages and apocrine cells. Although it is difficult to diagnose JP solely by its cytology, a combination of clinical and cytological findings may facilitate with the diagnosis of JP. There is no evidence to associate hormonal agent use or reproductive history with the occurrence of JP in young individuals, nor to associate JP with the maternal use of teratogenic agents during pregnancy (5).

Various breast disorders in children and young adults must be distinguished from JP. Rosen (6) described the rare types of papillary duct hyperplasia, which are observed in adolescence, including papilloma, papillomatosis and sclerosing papillomatosis. The most common symptom of papillary duct hyperplasia was the presence of a mass, although certain cases also exhibited nipple discharge, or presented with nipple discharge alone; however, all of these lesions lacked the cystic component that is characteristic of JP (6,17). Breast cancer is rare in children, however, when it does occur it most commonly takes the form of a secretory carcinoma and presents as a long-standing breast mass, which is occasionally painful (18). Nipple discharge was rarely identified. Secretory breast cancer is characterized by the presence of abundant intracellular and extracellular secretions, and intracytoplasmic vacuoles (19). Furthermore, immunoperoxidase staining for α-lactalbumin is typically positive in secretory carcinoma, but negative in JP (18). However, Rosen *et al* (5) reported a case of secretory carcinoma arising from JP and a case of JP with contralateral secretory carcinoma, therefore, the association between JP and secretory breast carcinoma appears likely and requires investigation.

Previous studies have demonstrated a relatively strong association between JP and breast cancer, particularly when there is a family history of breast cancer. Rosen *et al* (5) reviewed 84 cases of JP in 1982 and identified that 26% of the patients had a family history of breast cancer in at least one female relative. The majority of breast cancer cases were observed in older, secondary relatives (for example grandmothers or great aunts), although instances of maternal breast carcinoma were also reported. This may have been due to the young age of the JP patients and, therefore, the patients’ mothers or young female relatives may not have reached the peak age of breast cancer incidence. This is supported by a follow-up study of JP patients by Rosen and Kimmel in 1990 (8), in which 58% of cases had a family history of breast cancer, with mothers and maternal aunts exhibiting the highest risk. Additionally, Bazzocchi *et al* (7) observed that 33% of JP patients had a family history of breast cancer. These findings indicate that JP may be a marker of breast cancer in the family of the JP patients, thus, a thorough medical follow-up is recommended for JP patients and their families. Furthermore, microscopic evaluation revealed that breast carcinoma coexisted with JP in certain cases. Bazzocchi *et al* (7) identified that 15% of the JP patients presented with a coexisting carcinoma and Rosen *et al* (5) described three cases of other types of cancer coexisting with JP (n=84). Two of the patients exhibited secretory carcinoma (one arising from JP and another with contralateral secretory cancer) and the two patients had a maternal history of breast cancer. In addition, although the follow-up data was insufficient, the JP patients appeared to be at an increased risk of developing breast cancer. A previous study of 41 patients with a median follow-up period of 14 years demonstrated a 10% incidence of subsequent breast carcinoma in patients with JP (8). Although the risk of breast cancer should not be exaggerated, patients exhibiting any one of the following characteristics should be closely monitored for the subsequent development of breast cancer: i) Positive family history of breast cancer; ii) atypical proliferative lesions; iii) bilateral lesions; iv) multifocal lesions; or v) recurrence of JP. Patients with a positive family history of breast cancer and recurrent bilateral JP are considered to be at the greatest risk. However, due to the young age at which JP was diagnosed, the majority of the patients identified in the current study had not reached the peak age of incidence of breast cancer (range, 50–70 years). Further studies with a longer follow-up period are required to determine the incidence of breast cancer in patients with JP.

In conclusion, the recommended treatment strategy for JP is complete excision of the cancerous lesion to reduce local recurrence. On consideration of the current literature, it is prudent to advise an annual clinical follow-up, including a physical examination and/or ultrasonography of the breasts for JP patients, and for the patients’ female relatives, particularly those with a family history of breast cancer and with recurrent or bilateral JP.
